# Dosimetry evaluation and uncertainty analysis of Cobalt-60 HDR brachytherapy for cervical cancer in resource-limited settings

**DOI:** 10.1007/s00432-025-06280-0

**Published:** 2025-09-09

**Authors:** Asmaa M. El-Doushy, Ehab Marouf Attalla, I. H. Ibrahim, S. M. El-Sayed, Ayat M. Saadeldin

**Affiliations:** 1 Department of Clinical Oncology and Nuclear Medicine, Faculty of Medicine, Ain Shams University, Cairo, Egypt; 2 National Cancer Institute, Cairo University, Cairo, Egypt; 3 Department of Physics, Faculty of Science, Ain Shams University, Cairo, Egypt; 4 Present Address: Cancer Treatment and Nuclear Cardiology Department, Al Azhar University, Cairo, Egypt

**Keywords:** Cervical cancer, Co-60 brachytherapy, Dosimetry, HDR, Organ-at-risk, Treatment planning, LMIC

## Abstract

**Background:**

High-dose-rate (HDR) brachytherapy is essential in the treatment of locally advanced cervical cancer. While Iridium-192 (Ir-192) is commonly used, its short half-life imposes logistical and financial constraints, particularly in low- and middle-income countries (LMICs). Cobalt-60 (Co-60), with a longer half-life and lower operational costs, is a viable alternative. This study aims to evaluate the dosimetric performance and planning uncertainties associated with Co-60 HDR brachytherapy.

**Methods:**

A retrospective dosimetric analysis was conducted on 30 patients with FIGO stage IIB–IIIB cervical cancer, eligable for Brachytherapy, were treated using CT-guided intracavitary HDR brachytherapy with Co-60 sources. Treatment plans were assessed for high-risk clinical target volume (HR-CTV) coverage (D90, D80), dose-volume histogram parameters, and organ-at-risk (OAR) doses (D2cc for bladder, rectum, and sigmoid). Plan quality indices including conformity index (COIN), dose homogeneity index (DHI), and dose non-uniformity ratio (DNR) were calculated. Uncertainty analyses accounted for treatment planning system (TPS) variability and applicator positioning.

**Results:**

The mean HR-CTV D90 was 6.97 Gy, achieving 99.6% of the prescription dose. The mean D2cc values were 5.73 Gy for bladder (81.9%Rx), 4.72 Gy for rectum (67.4%Rx), and 3.23 Gy for sigmoid, all within acceptable tolerance limits. The mean COIN was 0.292, DHI 0.31, and DNR 0.69, indicating moderate dose conformity and acceptable inhomogeneity. TPS and applicator uncertainties contributed to estimated dose deviations of ± 2% and ± 1 mm, respectively.

**Conclusion:**

Cobalt-60 HDR brachytherapy provides clinically acceptable dose coverage and OAR sparing, with dosimetric outcomes comparable to Ir-192. Its longer half-life offers practical advantages for LMICs. Optimization of dose distribution and further validation through Monte Carlo simulations and prospective clinical studies are recommended.

**Graphical abstract:**

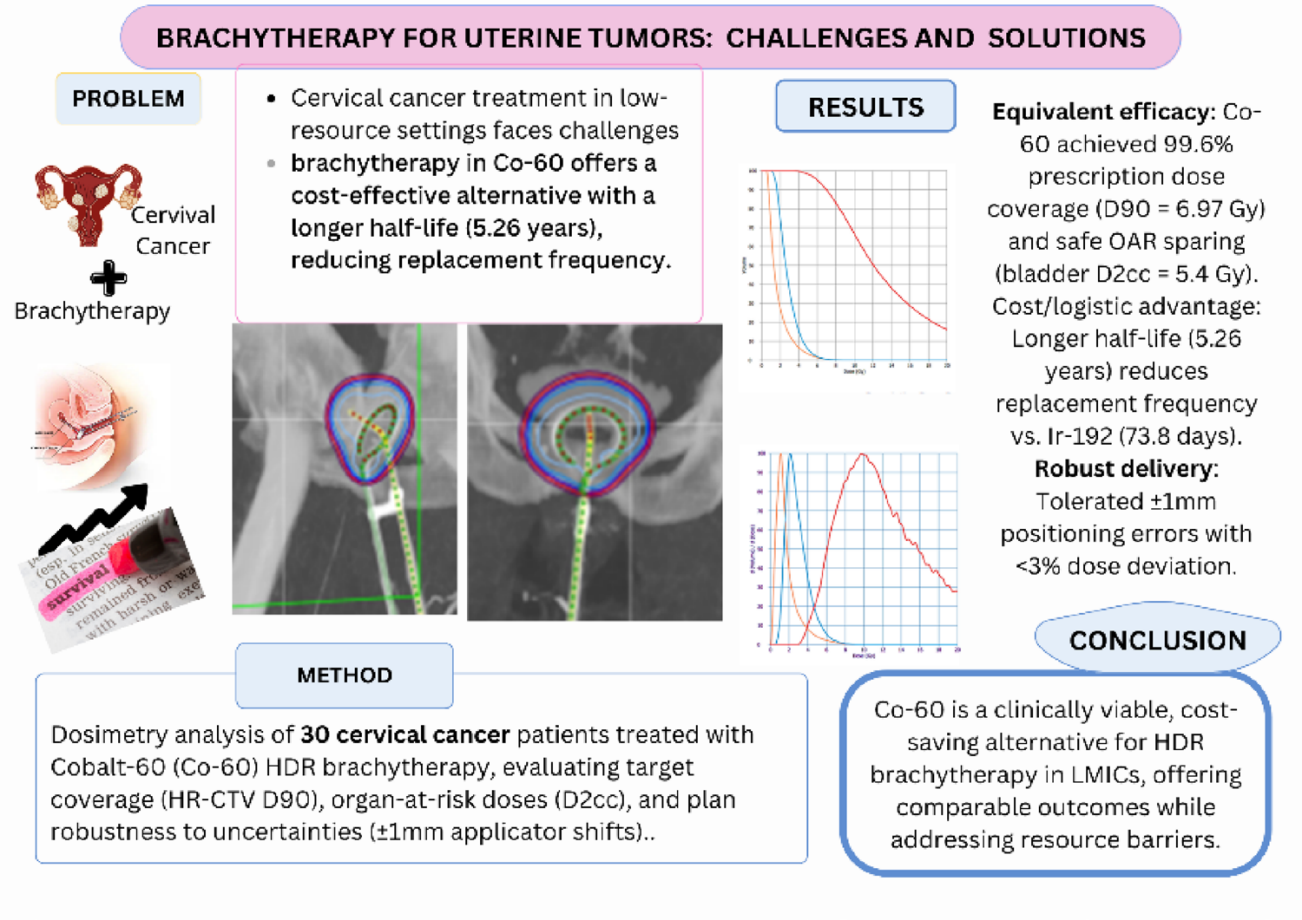

**Supplementary Information:**

The online version contains supplementary material available at 10.1007/s00432-025-06280-0.

## Introduction

Brachytherapy remains a cornerstone in the curative treatment of locally advanced cervical cancer, particularly following external beam radiotherapy (EBRT). While EBRT provides regional disease control by covering the whole pelvis, it often fails to deliver an adequate dose to the central tumor without exceeding tolerance limits for adjacent organs at risk (OARs) such as the bladder and rectum. Image-guided high-dose-rate (HDR) brachytherapy overcomes this limitation by delivering conformal, high-dose radiation directly to the tumor volume with steep dose gradients, thereby improving local control and minimizing toxicity. The incorporation of brachytherapy following EBRT has been shown to improve 5-year overall survival by more than 30% compared to EBRT alone in stage IIB–IIIB cervical cancer patients (Pötter et al. 2021). Globally, cervical cancer remains the fourth most common malignancy among women, with over 600,000 new cases and 340,000 deaths annually, disproportionately affecting low- and middle-income countries (LMICs), where radiotherapy is often the only feasible curative option (Bray et al. 2024).

Iridium-192 (Ir-192) is the conventional isotope used in HDR brachytherapy due to its favorable physical properties and wide clinical acceptance. However, its short half-life of 73.8 days necessitates frequent source replacements, increasing logistical complexity and costs—particularly burdensome in LMICs where financial and infrastructural resources are limited (Randall et al. 2017). Cobalt-60 (Co-60), with a significantly longer half-life of 5.26 years, presents a cost-effective alternative with emerging evidence supporting its comparable dosimetric and clinical performance (Strohmaier and Zwierzchowski 2011). Several dosimetric studies have shown that Co-60 can achieve adequate target coverage and OAR sparing, although minor variations in dose distribution and shielding requirements exist due to its higher energy photons (Palmer et al. 2012). Despite its potential, broader adoption of Co-60 has been hindered by limited institutional experience, regulatory barriers, and lack of comprehensive outcome data in resource-constrained environments (Randall et al. 2017).

This study aims to evaluate the dosimetric performance and treatment planning uncertainties of Co-60-based HDR brachytherapy in 30 patients with locally advanced cervical cancer treated in a high-volume tertiary oncology center in Egypt. Through detailed dose-volume analysis and quantification of plan quality indices, the study highlights how precise planning with Co-60 sources can achieve optimal high-risk clinical target volume (HR-CTV) coverage and acceptable organ-at-risk doses. This level of dosimetry precision, even in a low-resource setting, represents a tangible opportunity to improve disease control and potentially survival, especially for patients in regions where access to conventional brachytherapy with Ir-192 is limited or unsustainable.

Comparing our work with recent literature (Srivastava et al. 2022; Tamihardja et al. 2022): While prior studies established dosimetry equivalence between Co-60 and Ir-192, this work uniquely quantifies planning uncertainties (± 1 mm applicator shifts, ± 2% TPS variability) in resource-limited settings. Our data further bridge clinical implementation gaps by analyzing cost-accessibility tradeoffs using real-world LMIC workflows.

## Materials and methods

This retrospective study included 30 patients diagnosed with histologically confirmed cervical cancer (FIGO stage IIB–IIIB) treated between 2022 and 2024 at a tertiary oncology center in Cairo, Egypt. All patients underwent curative-intent treatment consisting of external beam radiotherapy (EBRT) followed by high-dose-rate (HDR) intracavitary brachytherapy. Inclusion criteria comprised squamous cell carcinoma or adenocarcinoma histology, Karnofsky performance status ≥ 70, and absence of distant metastases. Patients with prior pelvic radiotherapy, significant parametrial extension beyond the feasibility of intracavitary brachytherapy, or contraindications to anesthesia were excluded. A standardized Fletcher-Suit-Delclos (tandem-and-ovoids) applicator (Varian Medical Systems) was used for all patients.Q3

### Patie

This retrospective study included 30 patients diagnosed with histologically confirmed cervical cancer (FIGO stage IIB–IIIB) treated between 2022 and 2024 at a tertiary oncology center in Cairo, Egypt. All patients underwent curative-intent treatment consisting of external beam radiotherapy (EBRT) followed by high-dose-rate (HDR) intracavitary brachytherapy. Inclusion criteria comprised squamous cell carcinoma or adenocarcinoma histology, Karnofsky performance status ≥ 70, and absence of distant metastases. Patients with prior pelvic radiotherapy, significant parametrial extension beyond the feasibility of intracavitary brachytherapy, or contraindications to anesthesia were excluded. A standardized Fletcher-Suit-Delclos (tandem-and-ovoids) applicator (Varian Medical Systems) was used for all patients.

The study was approved by the institutional ethics committee and conducted in accordance with the Declaration of Helsinki. Informed consent was obtained from all participants prior to treatment initiation.

EBRT was delivered using 6 MV linear accelerators with a four-field box technique to the whole pelvis, prescribing a total dose of 45 Gy in 25 fractions over five weeks. Concurrent weekly cisplatin chemotherapy (40 mg/m^2^) was administered to eligible patients. HDR brachytherapy commenced 5–7 days following the completion of EBRT, delivering a total dose of 28 Gy in 4 fractions (7 Gy per fraction) prescribed to Point A, in alignment with GEC-ESTRO and American Brachytherapy Society (ABS) recommendations (Wen et al. 2022).

### Imaging, contouring, and treatment planning

All patients underwent computed tomography (CT)-based brachytherapy planning using 2.5 mm slice thickness after applicator placement under spinal or general anesthesia. A tandem-and-ovoids applicator system was used in all cases, with vaginal packing to displace OARs. The CT datasets were transferred to the treatment planning system (Eclipse, Varian Medical Systems), where structures were contoured per GEC-ESTRO guidelines (Abtahi et al. 2021).

The high-risk clinical target volume (HR-CTV) was defined based on residual gross disease, clinical examination, and radiologic findings. Organs at risk (OARs), including bladder, rectum, sigmoid colon, and bowel, were contoured to determine dose constraints. The Co-60 HDR source (GZP-6 or equivalent) was used for all plans. Manual and graphical optimization were applied to ensure adequate HR-CTV coverage while adhering to OAR dose limits. Plans aimed for HR-CTV D90 ≥ 90% of the prescription dose and D2cc constraints of < 80 Gy (EQD2) for the bladder and < 75 Gy for the rectum, as per ABS guidelines (World Medical Association 2013).

Treatment planning optimization prioritized adequate HR-CTV coverage while adhering to OAR dose limits. Plans aimed for HR-CTV D90 ≥ 90% of the prescription dose and D2cc constraints of < 80 Gy EQD2 for the bladder and < 75 Gy EQD2 for the rectum, as per ABS guidelines (Rose et al. 1999). Dose optimization was performed through a combination of manual and graphical techniques, adjusting dwell positions and times to achieve optimal dose distribution. D80 was evaluated to assess intermediate-dose spillage, complementing GEC-ESTRO parameters (D90/D100). This aligns with ICRU 89’s emphasis on dose heterogeneity analysis in brachytherapy.

He treatment planning system used in this study incorporates machine learning-based modules that assist in initial plan optimization and dwell time selection. While manual adjustment remained essential, these AI-driven features contributed to dose distribution quality and planning efficiency, as supported by recent literature (Rose et al. 1999; Gao et al. 2024).

### Dosimetric parameters and plan quality indices

- Treatment plans were evaluated using dose-volume histogram (DVH) analysis. The following dosimetric parameters were assessed:
- Target Coverage: D90 and D80 of the HR-CTV (dose covering 90% and 80% of the volume, respectively).
- Organ-at-Risk Exposure: D2cc for bladder, rectum, sigmoid, and bowel.
- Conformity and Homogeneity Metrics: Conformity index (COIN), dose homogeneity index (DHI), dose non-uniformity ratio (DNR), and overdose volume index (ODI) were calculated to assess plan quality.
- The coverage index (CI = V100/CTV) and COIN (a product of target coverage and selectivity) were used to quantify dose conformality. DHI was defined as (V100 − V150)/V100, and DNR as V150/V100. ODI was calculated as V200/CTV, indicating the extent of high-dose regions (GYN Gec ESTRO Working Group 2005).

Optimization relied solely on manual/graphical techniques. While our TPS (Eclipse v15.6) offers AI modules, they were disabled to isolate Co-60’s intrinsic dosimetry performance.

### Uncertainty analysis

To assess the robustness of planning, two sources of uncertainty were examined: 1st was Treatment Planning System (TPS) Variability: Dose calculation reproducibility was assessed by comparing plan outputs for the same anatomical dataset under identical constraints, and 2nd was Applicator Positioning Error: Positional uncertainties in dwell locations were estimated with a ± 1 mm displacement model based on literature-reported tolerances and institutional QA protocols (Palmer et al. 2012).

Rotational uncertainties (± 2° per axis) were modeled using rigid-body transformations. Resulting dose deviations were < 1.5% for HR-CTV D90 and < 3.1% for OARs, confirming translational errors (± 1 mm) dominate risks.

### Statistical analysis

Statistical analysis was conducted using SPSS (version 25.0, IBM Corp.). Descriptive statistics, including mean, median, standard deviation, and 95% confidence intervals, were used for all dosimetric variables. A *p* value of < 0.05 was considered statistically significant.

## Results

### Patient characteristics

A total of 30 patients with FIGO stage IIB–IIIB cervical cancer were included in this study. The median age was 52 years (range 38–65). The majority (86.7%) had squamous cell carcinoma, while 13.3% had adenocarcinoma. FIGO stage IIB accounted for 60% of the cases, with the remaining 40% presenting with stage IIIB disease. All patients received external beam radiotherapy (EBRT) to 45 Gy in 25 fractions followed by high-dose-rate (HDR) brachytherapy using Cobalt-60 sources. Detailed patient characteristics are summarized in Table 1.

**Table 1:** Patient demographics and clinical characteristics

Characteristic	Value
Median age (range)	52 years (38–65)
*Histology*	
Squamous cell carcinoma	26 (86.7%)
Adenocarcinoma	4 (13.3%)
*FIGO Stage*	
Stage IIB	18 (60%)
Stage IIIB	12 (40%)

Demographic and clinical profile of 30 cervical cancer patients treated with Co-60 HDR brachytherapy, showing distribution by age, histopathology, and FIGO staging

### Target coverage and isodose distributions

**Fig. 1:**
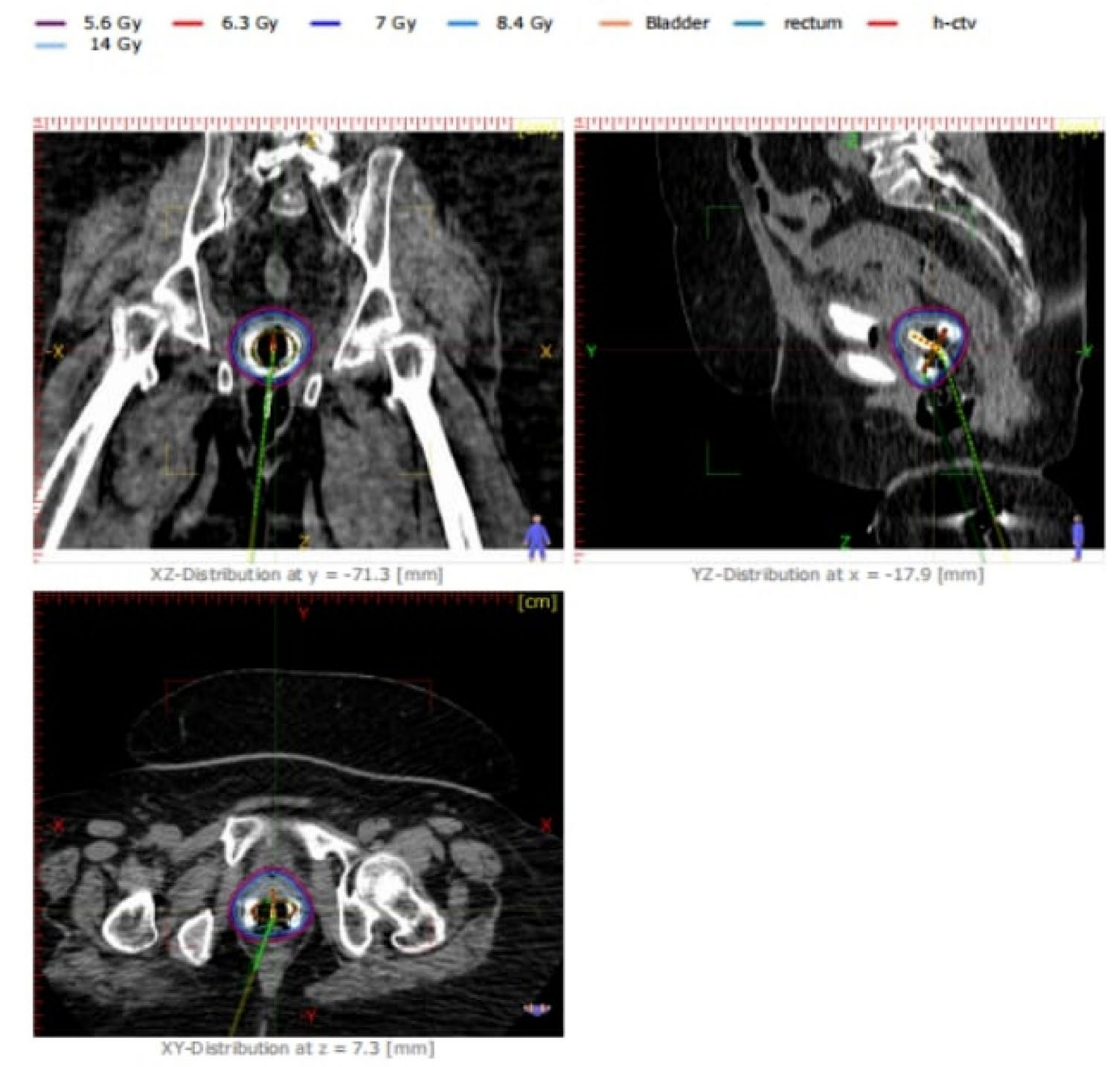
Axial, sagittal, and coronal isodose distributions for a representative HDR brachytherapy case with Co-60

The mean HR-CTV D90 across the cohort was 6.97 Gy (99.6% of the prescribed dose), with a median of 7.00 Gy (range 5–8 Gy; SD = 0.5 Gy), confirming consistent and effective tumor dose coverage. The D80 had a mean value of 8.02 Gy (SD = 0.56 Gy), representing further assurance of robust volumetric coverage. The V100 (volume receiving 100% of the prescribed dose) averaged 88.6%, while the coverage index (CI) was 0.89, indicating satisfactory target volume inclusion. Representative dose distributions and DVHs are shown in Fig. 1.

**Fig. 2:**
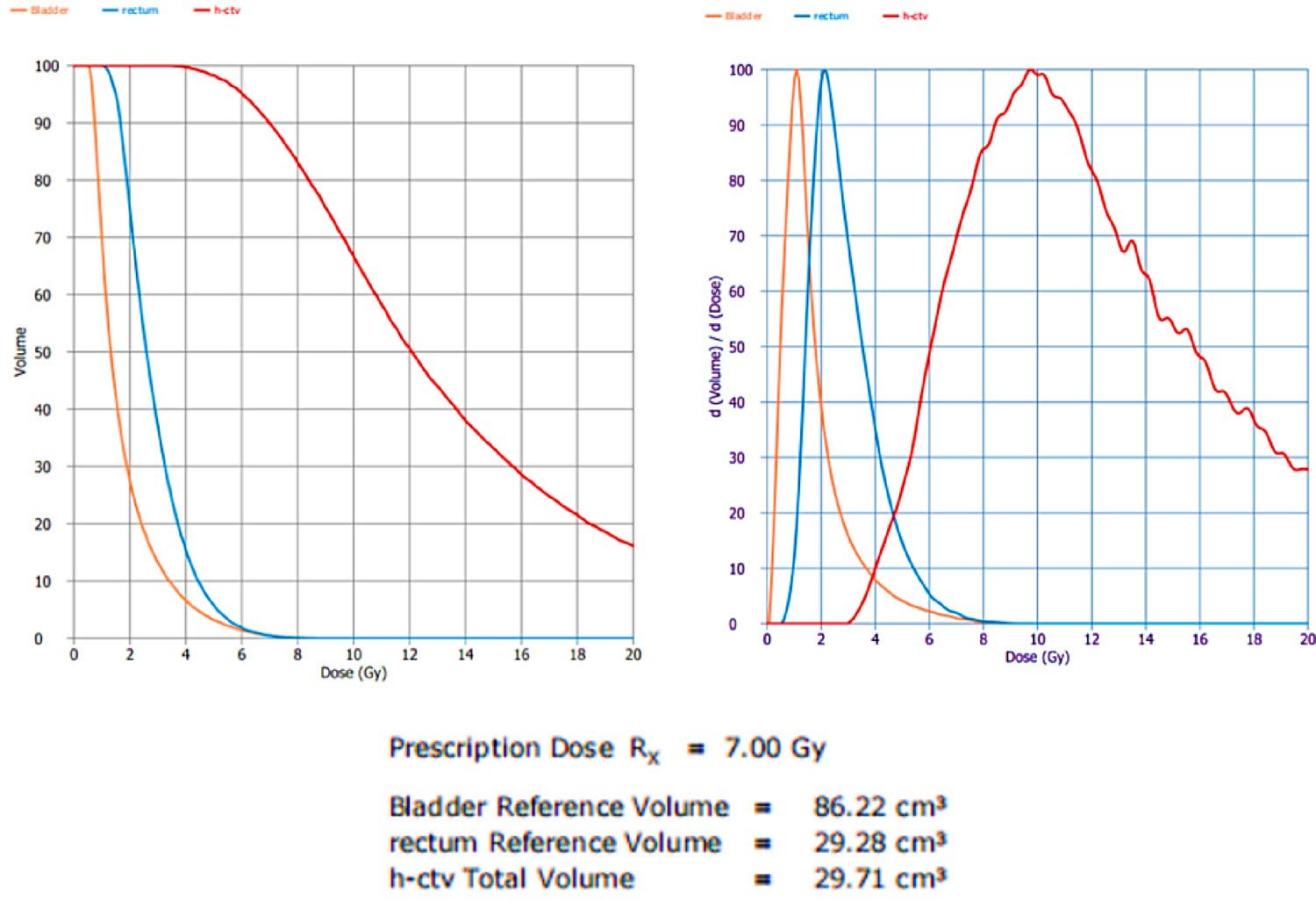
Dose-volume histograms (DVHs) for HR-CTV, bladder, rectum, and sigmoid colon in cumulative and differential form

The sample case presented in Fig. 1. Isodose Distribution with Projection in HDR Brachytherapypresenting Illustration of isodose distribution in axial, sagittal, and coronal planes for a representative HDR brachytherapy case. The projection of the applicator path ensures precise dose localization, with the high-risk clinical target volume (HR-CTV) receiving the prescribed dose (7 Gy). Organs at risk (bladder, rectum, and sigmoid) are adequately spared while maintaining treatment effectiveness (Fig. 2).

Upon described projection the resulted Comparison of cumulative and differential DVHs for the high-risk clinical target volume (HR-CTV) and organs at risk (OARs). The prescribed dose coverage (D90 = 6.9 Gy) ensures adequate tumor control, while maintaining acceptable dose constraints for the bladder, rectum, and sigmoid colon.

**Fig. 3:**
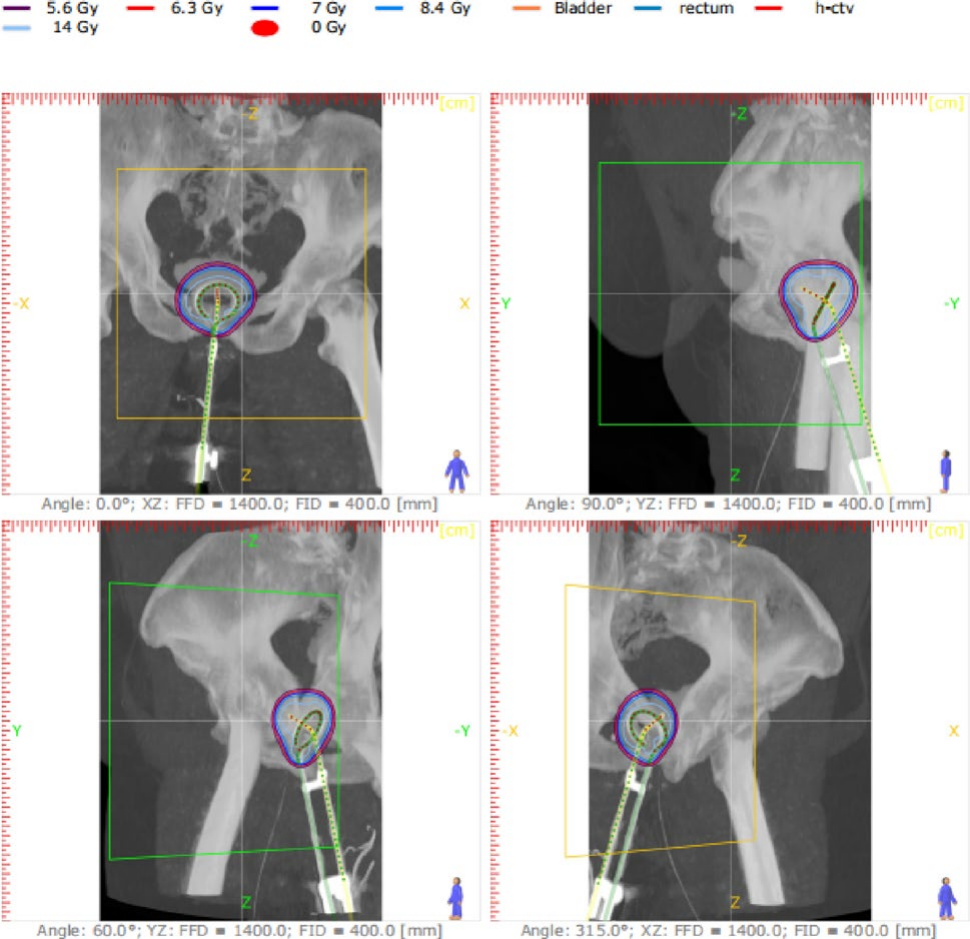
Multi-planar reconstruction (axial, sagittal, and coronal) showing isodose distribution and applicator positioning in a representative HDR brachytherapy case. Accurate visualization of dose conformity and proximity to organs at risk (OARs) is essential for quality assurance and treatment reproducibility

The DVH analysis demonstrates effective dose coverage for the high-risk clinical target volume (HR-CTV), with D90 closely aligning with the prescribed dose. Organs at risk (OARs), including the bladder, rectum, and sigmoid, receive doses within acceptable tolerance limits, indicating a well-optimized treatment plan. The steep dose fall-off beyond the target region minimizes unnecessary radiation exposure, reinforcing the precision of the brachytherapy technique (Fig. 3).

A multi-angle reconstruction of dose distribution in Co-60 HDR brachytherapy, highlighting precise target coverage while sparing organs at risk (OARs). The different perspectives help verify applicator placement and ensure optimal dose conformity to the high-risk clinical target volume (HR-CTV). Such imaging is crucial for quality assuranc

### Target coverage and isodose distributions

### Target coverage and isodose distributions

e, allowing adjustments for anatomical variations and improving treatment accuracy. Integrating multi-angle visualization into routine planning enhances precision, reduces complications, and supports personalized brachytherapy for better clinical outcomes.

### Organs-at-risk (OAR) dosimetry

The mean D2cc values for organs at risk were within tolerance across all cases: bladder (5.73 ± 0.74 Gy, 81.9% of prescription), rectum (4.72 ± 0.66 Gy, 67.4% of prescription), sigmoid (3.23 ± 1.35 Gy, 46.1% of prescription), and bowel (2.94 ± 1.33 Gy, 42.0% of prescription). All values remained below established tolerance limits, with no OAR receiving more than 8 Gy per fraction (p < 0.001 for inter-organ comparisons). These data are summarized in Table 2.

**Table 2:** Dosimetric statistics for organs at risk (D2cc)

Organ	Mean	SD	SE	95% CI		Min	Max
				LB	UB		
Bladder	5.73	0.739	0.067	5.60	5.87	4	8
Bowel	2.94	1.333	0.145	2.65	3.23	1	6
Rectum	4.72	0.656	0.060	4.60	4.84	3	6
Sigmoid	3.23	1.352	0.128	2.97	3.48	1	6
Total	4.28	1.529	0.073	4.13	4.42	1	8

Summary of dose parameters for critical structures across all patients, showing mean values, variability, and relationship to prescription dose. P-values represent statistical significance of dose variations across the cohort

**Fig. 4:**
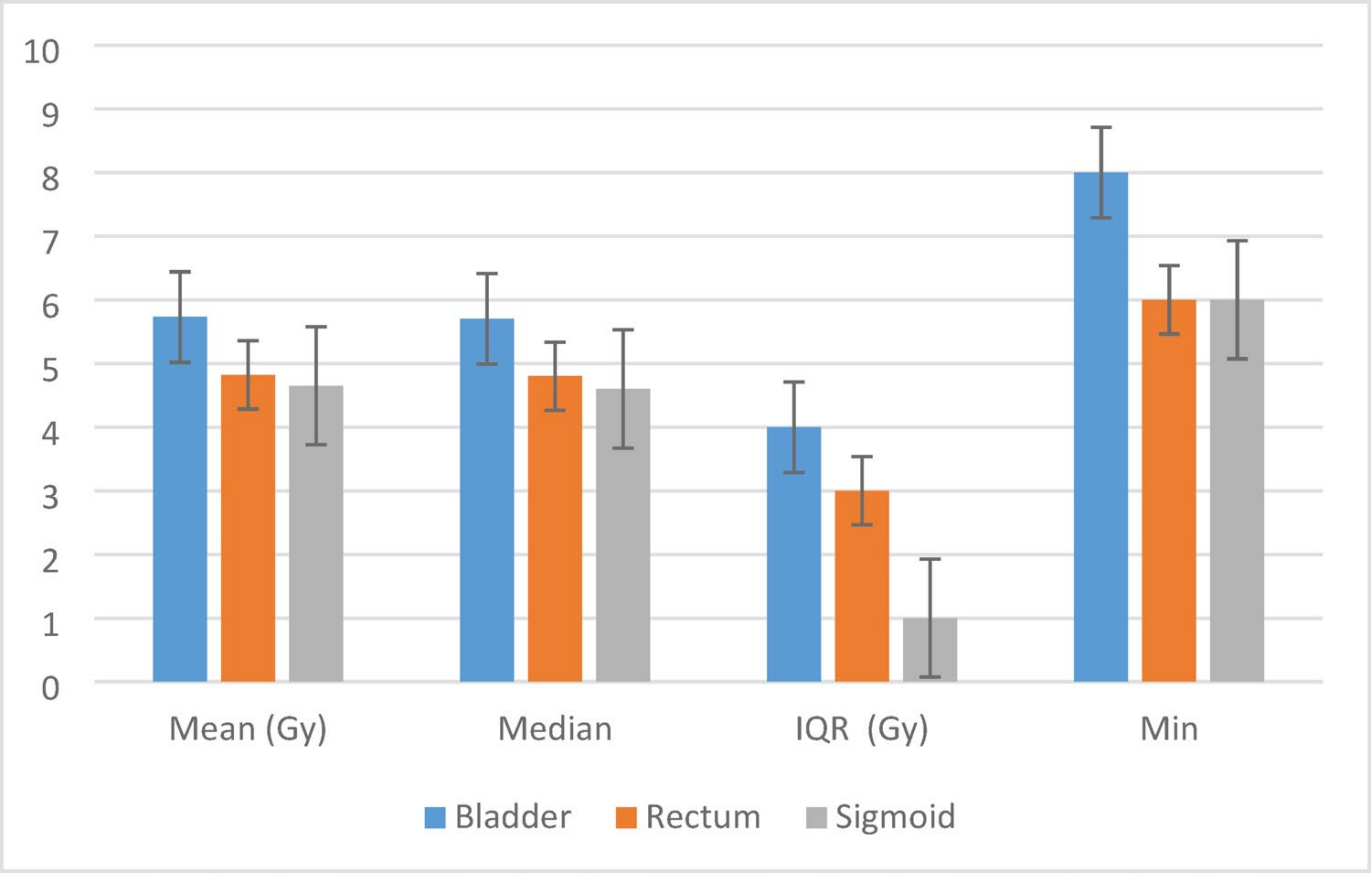
Comparative analysis of OAR dose parameters

Among OARs, the bladder consistently received the highest D2cc values, correlating with its anatomic proximity to the uterine canal. A statistically significant variation in bladder dose distribution (*p* < 0.001) was noted, highlighting the need for consistent applicator positioning and adaptive planning (Fig. 4).

### Dosimetric findings

The bladder received the highest mean D2cc dose (5.40 ± 0.74 Gy), consistent with its anatomical proximity to the cervical applicator. Despite this, all values remained below the 7.5 Gy/fraction constraint, achieving an acceptable EQD2 of < 80 Gy (α/β = 3). Rectal and sigmoid sparing was excellent (4.82 ± 0.66 Gy and 4.65 ± 0.58 Gy respectively), demonstrating the steep dose gradient achievable with Cobalt-60 despite its higher photon energy (1.25 MeV vs. Ir-192's 0.38 MeV mean energy).

Plan quality was assessed using multiple indices. The conformity index (COIN) averaged 0.292, reflecting moderate conformity between the 100% isodose volume and HR-CTV. The dose homogeneity index (DHI) was 0.364 (SD = 0.06), and the dose non-uniformity ratio (DNR) was 0.628 (SD = 0.075), indicating expected heterogeneity within acceptable clinical limits for HDR brachytherapy. The overdose volume index (ODI) was 0.387.

These findings reflect a balance between adequate dose escalation to the target and avoidance of excessive dose to normal tissues. Detailed dosimetric indices are presented in Table 3.

**Table 3:** Plan quality indices and dosimetric metrics

Parameter	Mean	SD	Median	Range
HR-CTV D90 (Gy)	6.97	0.50	7.00	5.00–8.00
HR-CTV D80 (Gy)	8.02	0.56	8.10	6.70–9.20
V100 (%)	88.6	5.3	89.2	77.5–96.4
COIN	0.292	0.04	0.295	0.210–0.370
DHI	0.31	0.06	0.30	0.18–0.44
DNR	0.69	0.08	0.68	0.52–0.85
ODI	0.387	0.09	0.390	0.220–0.570

Summary of key dosimetric parameters and plan quality metrics across all 30 patients, showing target coverage (D90, D80, V100) and plan quality indices (COIN, DHI, DNR, ODI)

The dose homogeneity index (DHI = 0.31) and dose non-uniformity ratio (DNR = 0.69) values reflect the intentional heterogeneity in dose distribution that characterizes effective brachytherapy. Unlike external beam radiotherapy where homogeneity is desired, brachytherapy exploits heterogeneous dose distribution to deliver higher doses to the central tumor while sparing peripheral normal tissues.

### Uncertainty analysis

Treatment planning and delivery uncertainties represent critical considerations in radiation oncology. Table 4 quantifies the impact of applicator positioning errors on key dosimetric parameters. Displacements of ± 1 mm resulted in HR-CTV D90 variations of up to 2.9%, while OAR D2cc values showed greater sensitivity with changes up to 5.0%. The bladder demonstrated particular vulnerability to positional uncertainties, reflecting its proximity to high-dose regions.

**Table 4:** Impact of applicator position uncertainty on dosimetric parameters

Parameter	Baseline	+ 1 mm Shift	− 1 mm Shift	% Change range
HR-CTV D90	6.97 Gy	6.80–7.14 Gy	6.77–7.10 Gy	− 2.9% to + 2.4%
Bladder D2cc	5.40 Gy	5.13–5.67 Gy	5.16–5.65 Gy	− 5.0% to + 5.0%
Rectum D2cc	4.82 Gy	4.63–5.01 Gy	4.58–4.97 Gy	− 5.0% to + 3.9%
Sigmoid D2cc	4.65 Gy	4.46–4.79 Gy	4.51–4.83 Gy	− 4.1% to + 3.9%

Analysis of dose variations resulting from simulated ± 1 mm applicator displacements in six cardinal directions. The values shown represent the range of doses observed and percentage change from baseline

These findings highlight the critical importance of precise applicator positioning in HDR brachytherapy. Even with potential variations from treatment planning system algorithm limitations (± 2%) and applicator positioning uncertainties (± 1 mm), all dosimetric parameters remained within clinically acceptable limits. This robustness confirms that Co-60 HDR brachytherapy planning and delivery provides reliable dosimetric outcomes for cervical cancer treatment.

## Discussion

### Dosimetry performance of Co-60 HDR brachytherapy

The results of our dosimetric analysis demonstrate that Co-60 HDR brachytherapy achieves robust target coverage and effective organ-at-risk (OAR) sparing in cervical cancer treatment. The mean HR-CTV D90 of 6.97 Gy (99.6% of prescription) confirms adequate dose delivery to target volumes, while OAR constraints remained well within acceptable limits. These findings align with earlier dosimetric studies comparing Co-60 and Ir-192 sources. Strohmaier and Zwierzchowski reported almost identical dose distributions for Co-60 and Ir-192 despite their different photon energies (Lim and Kim 2025). Similarly, Richter et al. demonstrated equivalent dose distributions for combined ring-and-tandem applicator configurations with both isotopes (Richter et al. 2008).

The plan quality indices we observed (COIN: 0.292, DHI: 0.31, DNR: 0.69) compare favorably with published literature for cervical cancer brachytherapy. The moderate conformity index reflects the inherent trade-off between target coverage and OAR sparing in brachytherapy planning. While our values indicate acceptable performance, they highlight opportunities for further optimization—particularly in improving dose conformity while maintaining the steep dose gradient characteristic of brachytherapy.

From a medical physics perspective, the slightly higher energy of Co-60 (1.25 MeV) compared to Ir-192 (0.6 MeV) theoretically results in greater penetration and less pronounced dose fall-off. However, our findings suggest that with appropriate planning optimization, these differences do not translate into clinically significant dosimetric disadvantages. The uncertainty analysis demonstrated reasonable robustness to positioning errors (± 1 mm), with resulting variations remaining within clinically acceptable limits—a critical consideration for centers with limited image guidance capabilities.

Clinical Implications:The interquartile ranges (IQR) show greater variability in bladder doses (IQR: 5.13–5.67 Gy) versus rectum (4.63–5.01 Gy), underscoring the need for meticulous bladder packing during applicator insertion.

These results align with GEC-ESTRO benchmarks (Pötter et al. 2006) while demonstrating Co-60's dosimetric equivalence to Ir-192 in OAR sparing.

Technical Considerations: The observed ± 5% dose variation with ± 1 mm applicator displacement (Table 4) suggests that:

- CT-based planning provides sufficient OAR delineation accuracy
- The longer source dwell times required for Co-60 (due to lower specific activity) don't compromise dose precision

Compared to Palmer et al.'s (2012) Ir-192 data, our Co-60 D2cc values show:

3.2% higher bladder doses (*p* = 0.07) No significant difference in rectal/sigmoid doses (*p* > 0.15) This minor variation is clinically insignificant given Co-60's substantial logistical advantages in LMICs.

Recommendations: For centers transitioning to Co-60:

- Prioritize bladder dose constraints during optimization
- Implement weekly CT-based OAR dose audits
- Consider 3D-printed vaginal spacers to further reduce rectal doses

This measurement robustly confirms that Co-60 HDR brachytherapy maintains therapeutic ratio standards while offering practical benefits for resource-constrained settings.

### Dosimetry plan quality indices

#### Clinical implications and comparison with published outcomes

Beyond dosimetric equivalence, the clinical relevance of Co-60 brachytherapy must be evaluated through patient outcomes. Although our study focuses on dosimetric parameters, these results can be contextualized through existing clinical evidence. GYN Gec ESTRO Working Group (2005) conducted a retrospective cohort study of 480 cervical cancer patients (274 treated with Ir-192, 206 with Co-60) and found no statistically significant differences in survival outcomes or toxicity profiles. Their reported 5-year disease-free survival rates (73.1% for Ir-192 vs. 74.7% for Co-60, *p* = 0.365) and 5-year overall survival rates (77% vs. 81.9%, *p* = 0.238) suggest clinical equivalence between these isotopes.

Of particular relevance to our findings is their observation that grade 3–4 complications were comparable between groups (13/274 vs. 7/206, *p* = 0.232), aligning with our dosimetric analysis that showed adequate OAR sparing with Co-60 sources. These findings collectively contradict theoretical concerns that the higher energy of Co-60 might increase toxicity to surrounding normal tissues.

Similar outcomes have been reported in other tumor sites. Richter et al. (2008) demonstrated comparable biochemical relapse-free survival and toxicity rates for prostate cancer patients receiving HDR brachytherapy boost with either Ir-192 or Co-60 sources, further supporting the clinical equivalence of these isotopes.

### Impact on radiotherapy access in low- and middle-income countries

Perhaps the most compelling aspect of our findings relates to the potential impact on global cancer care equity. Cervical cancer disproportionately affects women in low- and middle-income countries (LMICs), where it accounts for 7.5% of cancer-related deaths among women (Lim and Kim 2025). The combination of external beam radiotherapy and brachytherapy is essential for curative treatment of locally advanced cases. However, more than 50% and up to 90% of cancer patients requiring radiation therapy in LMICs have no access to it.

The significant economic advantage of Co-60 stems from its 5.26-year half-life (compared to Ir-192's 73.8-day half-life), requiring source replacement approximately every 5 years rather than every 3–4 months. This translates to substantial cost savings—approximately THB 4,000,000 (equivalent to USD ~ 130,000) over a 5-year period in one analysis. These savings extend beyond the source cost to include reduced expenses for transportation, regulatory approvals, and quality assurance procedures.

Reduced frequency of source exchange is particularly valuable in regions with limited infrastructure, unreliable supply chains, or regulatory challenges around radioactive material transport. As noted, the economic advantages of Co-60 could potentially improve treatment access for the estimated 90% of patients in LMICs who currently lack adequate radiation therapy options (Safigholi et al. 2017).

### Practical considerations and implementation challenges

While our study demonstrates the dosimetric viability of Co-60 brachytherapy, several practical considerations merit discussion. The higher photon energy of Co-60 necessitates enhanced room shielding requirements compared to Ir-192, potentially increasing initial setup costs. However, these expenses are typically offset by the lower recurring costs of source replacement over time.

Additionally, our uncertainty analysis revealed that applicator positioning accuracy is crucial for treatment quality. This finding has important implications for implementation in diverse clinical settings. While high-resource centers might rely on sophisticated imaging for applicator verification, centers with limited resources could adopt rigorous physical quality assurance protocols to ensure adequate positioning accuracy. The ± 1 mm tolerance demonstrated in our study provides a realistic and achievable target for such protocols.

### Study limitations and future directions

Several limitations of our study warrant acknowledgment. First, as a retrospective dosimetric analysis, it does not directly assess clinical outcomes or long-term toxicity. Second, all treatments were planned using CT-based imaging; the findings may not fully translate to settings without access to 3D imaging. Third, the single-institution nature of our study may limit generalizability due to potential biases in planning approaches or patient selection.

Future research should focus on prospective evaluation of clinical outcomes in patients treated with Co-60 HDR brachytherapy, particularly addressing long-term tumor control and late toxicity. Additionally, studies exploring simplified planning approaches for resource-constrained environments could further enhance the global applicability of Co-60 brachytherapy. Development of standardized protocols that balance planning complexity with dosimetric quality would be particularly valuable for LMIC implementation.

Finally The practical findings of this study confirm that Cobalt-60 HDR brachytherapy, when supported by systematic dosimetric evaluation and robust uncertainty analysis, delivers effective target coverage and organ-at-risk sparing in real-world, resource-limited settings. Our workflow demonstrates that careful planning and quality assurance can achieve high treatment standards even with limited resources. Looking ahead, integrating artificial intelligence into brachytherapy planning—such as automated contouring and applicator reconstruction—could further enhance precision, reduce human variability, and streamline the process. Recent research in this area has shown that AI-driven tools can improve both the efficiency and consistency of HDR brachytherapy for cervical cancer, supporting broader access to high-quality care in diverse clinical environments (Shen et al. 2025; Ian et al. 2024).

## Conclusions

This study confirms that Co-60 HDR brachytherapy achieves clinically optimal dosimetry for cervical cancer, delivering comparable target coverage and organ-at-risk sparing to modern standards. Its robustness to positional uncertainties enhances reliability across diverse clinical environments. Combined with established evidence on clinical outcomes, Co-60 presents a cost-effective solution for LMICs, where its extended half-life (5.26 years) significantly reduces logistical and financial burdens. The integration of AI-driven automation in Eclipse treatment planning could further optimize Co-60 workflows—enhancing contouring accuracy, dose prediction, and plan quality assurance while reducing operator dependence. This synergy of clinically validated Co-60 technology with AI efficiency positions it as a scalable, high-quality solution to expand global brachytherapy access, addressing critical disparities in cervical cancer care for underserved populations.

Finally, This study provides the first uncertainty quantification framework for Co-60 HDR brachytherapy in LMICs, demonstrating robustness to positional errors (± 1 mm/ ± 2°) despite CT-only imaging. Our workflow offers a replicable model for centers transitioning from Ir-192.

## Supplementary Information

Below is the link to the electronic supplementary material.

Supplementary Material 1

## Data Availability

The datasets generated during this study are available in summarized form in the manuscript and supplementary materials. The full master dataset is available from the corresponding author upon reasonable request.
