# Supplementary figures and images for: Dosimetry evaluation and uncertainty analysis of Cobalt-60 HDR brachytherapy for cervical cancer in resource-limited settings
Source: J Cancer Res Clin Oncol. 2025 Sep 9;151(9):247. doi: 10.1007/s00432-025-06280-0 (PMC12420530; doi:10.1007/s00432-025-06280-0)

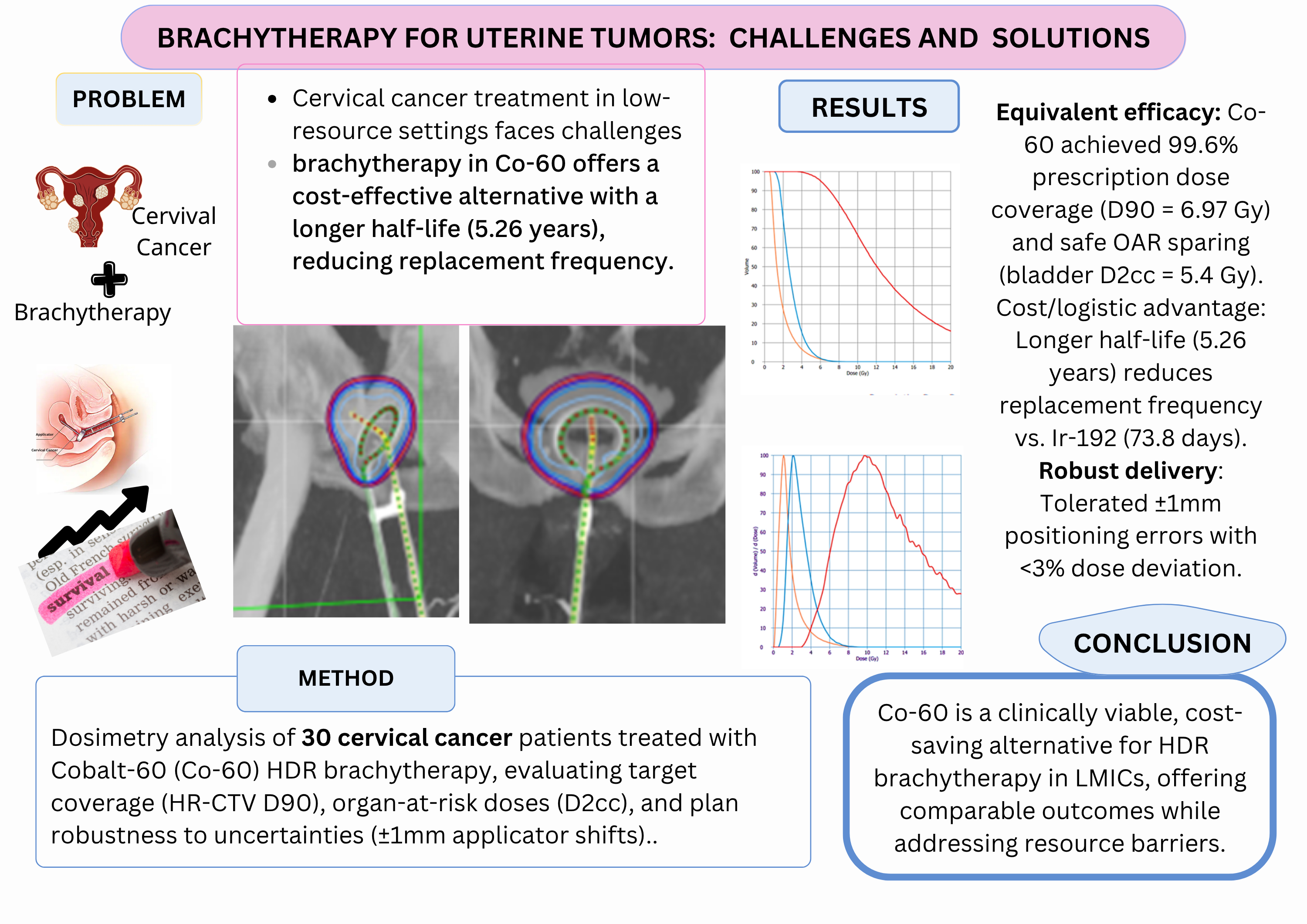

Supplement: Supplementary file 1 — Supplementary Material 1 [file 432_2025_6280_MOESM1_ESM.png]
